# Practical Observations on the Medical Powers of the Most Celebrated Mineral Waters, and of the Various Modes of Bathing. Intended for the Use of Invalids

**Published:** 1819-10-01

**Authors:** 


*Practical Observations on the Medical Powers of the most celebrated Mineral Waters, and of the various Modes of Bathing. Intended for the Use of Invalids.* By Patrick Mackenzie, M. D. Licentiate of the Royal College of Physicians; and Assistant Physician to the Fever Institution, &c. Small Octavo, pp. 152. London, 1819.

The title page of this work is a sufficient indication of its object; but the following passage, in the preface, presents an insuperable bar to the institution of any critical or analytical process relative to its merits, in a Journal exclusively devoted to medical literature.

"I have avoided all scrupulous chemical inquiries, and theoretical discussions, as incompatible with my object, confining myself to a plain statement of medical facts only, the work being intended for the valetudinarian, and not for the critical eye of general science, *nor of the medical profession.*"

Notwithstanding the foregoing rebuff, we have taken the liberty of glancing into the work; and the result has been, a conviction that it is by no means unworthy of a place in the general practitioner's library, since it conveys a concise description of the various mineral springs, arranged according to their medical operations, rather than their chemical qualities; and consequently offering a ready reference, when consulted by patients on the eve of an annual flight to some fashionable watering place.

